# Abdominal Aortic Aneurysm: Can the Anaconda™ Custom-Made Device Deliver? An International Perspective

**DOI:** 10.3389/fcvm.2022.959149

**Published:** 2022-07-14

**Authors:** Matti Jubouri, Abedalaziz O. Surkhi, Sven Z. C. P. Tan, Damian M. Bailey, Ian M. Williams, Mohamad Bashir

**Affiliations:** ^1^Hull York Medical School, University of York, York, United Kingdom; ^2^Faculty of Medicine, Al-Quds University, Jerusalem, Palestine; ^3^Barts and The London School of Medicine and Dentistry, Queen Mary University of London, London, United Kingdom; ^4^Neurovascular Research Laboratory, Faculty of Life Sciences and Education, University of South Wales, Cardiff, United Kingdom; ^5^Department of Vascular Surgery, University Hospital of Wales, Cardiff, United Kingdom; ^6^Vascular and Endovascular Surgery, Velindre University NHS Trust, Health Education and Improvement Wales (HEIW), Cardiff, United Kingdom

**Keywords:** abdominal aortic aneurysm (AAA), aneurysm, Anaconda, EVAR, fenestrated, custom-made

## Abstract

**Introduction:**

Since the introduction of endovascular aortic repair (EVAR), it has demonstrated excellent clinical outcomes and has replaced open surgical repair (OSR) in the treatment of abdominal aortic aneurysms (AAA). AAA is a life-threatening abnormal dilation of the abdominal aorta to 1.5 times its normal diameter. Several commercial EVAR devices exist on the global market, with the Terumo Aortic Fenestrated Anaconda™ graft showing superiority. In this study, we sought to provide an international perspective using multicenter-multinational data on the Anaconda™ device characteristics, design, and delivery, and discuss relevant literature.

**Materials and Methods:**

This study represents a cross-sectional international analysis of custom-made fenestrated Anaconda™ device. Ethical and legal approval for data collection was obtained from each of the local authorities. For the statistical analysis, SPSS 28 for Windows and R were utilized. Pearson’s chi-square analysis was used to assess differences in cumulative distribution frequencies between select variables. Statistical significance for all two-tailed tests was set at *p* < 0.05.

**Results:**

A total of 5,030 Anaconda™ devices were implanted during the 9-year study period in 27 countries spanning 6 continents. The predominant device category was bifurcate (83.6%), whereas the most common proximal ring stent configuration being standard (64.5%). All devices were delivered within 8 weeks of diagnosis, with most being implanted within 6–8 weeks (55.4%). The Anaconda™ was indicated in the 3,891 (77.4%) patients due to competitor rejection/inability to treat unsuitable/complex aortic anatomy. In the remaining 1,139 (22.6%) patients, it was utilized based on surgeon preference. Almost all devices (95%) were delivered along with a prototype. Of the total 5,030 Anaconda™ devices, 438 (8.7%) used 0–1 fenestrations, 2,349 (46.7%) used 2–3, while 2,243 (44.6%) utilized 4, 5, or 6 fenestrations.

**Discussion:**

The Terumo Aortic Fenestrated Anaconda™ device features a highly unique and innovative design that enables it to treat highly complex aortic anatomy while achieving excellent results. The Anaconda™’s custom-made approach allows it to be tailored to individual patient anatomy, in addition to the device prototype provided by Terumo Aortic optimize clinical outcomes. Finally, the fenestrated Anaconda™ is a highly versatile device offering a wide range of device categories, configurations, and sizes.

## Introduction

Abdominal aortic aneurysm (AAA) is a life-threatening abnormal dilation of the abdominal aorta to >3 cm, which is 1.5 times its normal diameter and can lead to serious complications such as rupture (1, 2). Importantly, ruptured AAA can have a mortality of up to 80% if untreated (3). Management of AAA includes repair, which can be performed either endovascularly or *via* open surgical repair (OSR). OSR was considered the mainstay treatment for AAA for many years; however, since the introduction of endovascular aortic repair (EVAR) almost three decades ago, it has seen major utilization in the treatment of AAA, replacing OSR for most elective cases (4–6). Considering lower mortality and improved morbidity, EVAR now accounts for 60% of elective infrarenal AAA repairs, 61.3% of complex AAA repairs, and 41.3% of ruptured AAA repairs in the United Kingdom since 2020 (7).

Several EVAR stent-grafts exist commercially on the global market, these having undergone significant upgrades over the years to widen their armamentarium in tackling increasingly complex AAA pathology and improve clinical outcomes (8). EVAR devices can be predominantly split into three main categories, namely, off-the-shelf endografts, physician-modified devices, or custom-made devices (CMDs), with their main variants being standard, fenestrated, and branched EVAR (9). The Anaconda™ AAA stent-graft system is a leading custom-made fenestrated EVAR (FEVAR) device developed by Terumo Aortic and has been associated with excellent clinical outcomes. The Anaconda™ device can be considered the superior commercial EVAR device on the market due to its unique design and high versatility with multiple device categories.

Furthermore, there are different configurations with a global market for its use. These are in addition to its global use, outstanding custom-made approach, and superior results (10–12).

In this study, we sought to provide an international perspective using multicenter-multinational data on the Anaconda™ device delivery, including its categories, configurations, geographical distribution, indications, delivery time frame, prototype requirement, and custom-made approach for fenestrations. Essentially, can the Anaconda™ CMD deliver?

## The Custom-Made Fenestrated Anaconda™: Design

The current third-generation Anaconda™ device used globally evolved from two earlier versions, first of which was introduced in 1998 to address some of the failures observed with other EVAR stent-grafts in the 1990’s (8, 10, 12). The first-generation Anaconda™ device did not feature any hooks and completely relied on friction sealing for proximal fixation and prevention of endograft migration. The second-generation device had independent nitinol rings, zero columnar support, and straight limbs (10). This device underwent modifications based on many years of experience to develop its current version. The third-generation Anaconda™ features the ONE-LOK™ platform (8). This readjustable self-expanding bimodular device comprised of a nitinol skeleton that supports a woven polyester fabric (13). The flexible skeleton is formed by independent nitinol stent rings in a circular design, with zero body support of the graft with the possibility of adding an augmented valley (8, 10, 12). This design has proven to have greater flexibility, as well as lower stress values than Z-stents such as Zenith, and thus better durability (14). The proximal seal is achieved with the two saddle-shaped proximal nitinol ring stents in addition to hooks connected to them, which is a new feature of the third-generation Anaconda™ added to absolutely minimize incidence of endograft migration. Three to four nitinol hooks support the proximal seal to the aortic wall (8, 10, 12). The device also features a magnetic guidewire/docking system for navigating the bifurcated distal portion of the graft and can be used for cannulation of the contralateral gate (15).

As for the limbs, these are supported with independent nitinol rings to prevent kinking and come in three configurations according to iliac artery diameter, namely, tapered, straight, and flared, each measuring either 60, 100, or 120 mm in length (10, 15, 16). One unique feature of the Anaconda™ is its high versatility, offering a wide range of body-limb size combinations, as the docking zone limb diameter was standardized in its third iteration (16). The main body of the graft is commercially available in eight different diameters ranging from 19.5 to 34 mm while the iliac leg branches are also available with diameters ranging from 9 to 18 mm (15). Once inserted, it can be fully repositioned by use of the control collar of the delivery system handle (12, 17). This feature is vital in FEVAR to ensure perfect alignment between fenestrations and target vessels ostia. In addition, the fenestrations can be cannulated from either end of the graft (17).

The Anaconda™ custom-made approach allows the device to be tailored to individual patient anatomy and expands its use for treating hostile aortic anatomy such as angulated or stenosed necks and diseased iliac artery branches (12, 13). However, this is only feasible for elective cases. Although Anaconda™ construction time is between 3 and 6 weeks, this is less than half the time required for other competitor devices to be customized (10–12 weeks) (12, 18). In addition, device prototypes are provided by Terumo Aortic. Preoperatively, a 3D model of the patient’s exact aorta can be printed, and the procedure can be rehearsed using the non-sterile device prototype (12). In turn, any modifications to the device or plan can be done at that point to allow for a safer smoother procedure and optimized results. Finally, the Anaconda™ instruction for use (IFU) recommends oversizing the stent-graft by 10–20% and indicates the possibility for treating highly angulated necks with indication for up to 90°, ensuring that clinicians are using the device on label (12, 17).

## Materials and Methods

### Study Design

A cross-sectional international multicenter analysis involving 27 countries was conducted to investigate the application of Anaconda™ endovascular stent-graft. The data were collected in a prospective manner between March 2013 and March 2022 and stored in a registry. Ethical approval for data collection was obtained from each of the local authorities. Geographically, European regions were classified according to the United Nations Geoscheme for Europe.

### Operative Characteristics

A total of 5,030 patients were treated for AAA using the Anaconda™ stent-graft. The decision to utilize Anaconda™ was based on two criteria. In 22.6% of cases (*n* = 1,139), the Anaconda™ was used according to surgeon preference. More importantly, the Anaconda was utilized in the majority of cases (77.4%; *n* = 3,891) due to competitor incompatibility with unsuitable/complex aortic anatomy. A total of 581 of the procedures (11.5%) had a procedural time of 90–110 min and an endovascular time of 60–75 min. A total of 4,463 of the procedures (88.2%) had a procedural time of 120–160 min and an endovascular time of 80–120 min. A 100% successful implantation rate was achieved in 99.9% of patients with the Anaconda™.

### Statistical Analysis

All statistical analyses were conducted using SPSS (IBM SPSS 28 for Windows) with the R plugin. The prospective data were analyzed in a retrospective manner. Descriptive statistics were performed, and comparative analyses were done on relevant variables. Pearson chi-square analysis was used to assess differences in cumulative distribution frequencies between select variables. Statistical significance for all two-tailed tests was set at *p* < 0.05.

## Results

### Device Categories

**FIGURE 1:**
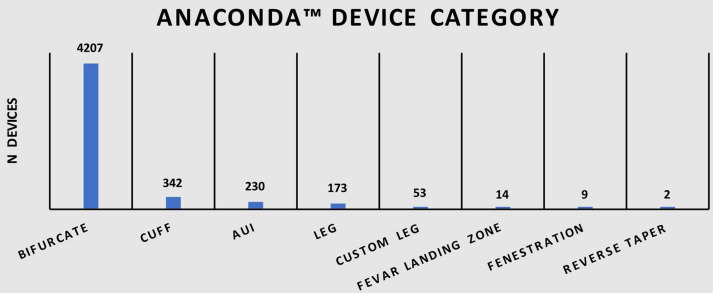
Illustration of Anaconda™ device categories.

A total of 5,030 Anaconda™ devices split over eight device categories were recorded in this study. As shown in Table 1, 83.6% (*n* = 4,207) were categorized as bifurcated devices, 6.8% (*n* = 342) as cuff devices, 4.6% (*n* = 230) as Aorto-uni-iliac (AUI) devices, 3.4% (*n* = 173) as leg devices, 1.1% (*n* = 53) as custom leg devices, 0.3% (*n* = 14) as FEVAR landing zone devices, 0.2% (*n* = 9) as fenestrated devices, and <0.1% (*n* = 2) as reverse taper devices. Figure 1 illustrates the distribution of devices among various categories.

**TABLE 1:** Summary of Anaconda™ device categories.

Anaconda™ device category	*n* = 5030 (%)
Bifurcate	4207 (83.6)
Cuff	342 (6.8)
AUI	230 (4.6)
Leg	173 (3.4)
Custom Leg	53 (1.1)
FEVAR Landing zone	14 (0.3)
Fenestration	9 (0.2)
Reverse Taper	2 (< 0.1)

### Geographical Distribution of Use

**FIGURE 2:**
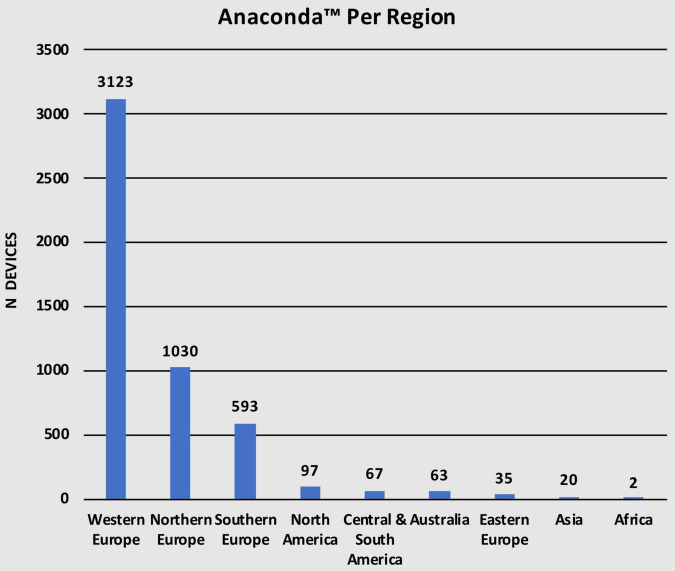
Geographical distribution of Anaconda™ utilization.

This study represents a multinational Anaconda™ registry spanning 27 countries on 6 continents, with the most common being Europe. Out of the total 5,030 Anaconda™ stent-grafts used over the 9-year period, 3,123 (62.1%) were used in Western Europe, 1,030 (20.4%) in Northern Europe, 593 (11.8%) in Southern Europe, and 35 (0.7%) in Eastern Europe. In addition, 97 (1.9%) devices were used in North America, 67 (1.3%) in Central and South America, 63 (1.2%) in Australia, 20 (0.4%) in Asia, and only 2 (< 0.1%) devices in Africa (Table 2). Figure 2 describes the number of devices in every region. European regions were divided according to the United Nations Geoscheme for Europe.

**TABLE 2:** Anaconda™ per geographical region.

Region	n (%)
Western Europe	3123 (62.1)
Northern Europe	1030 (20.4)
Southern Europe	593 (11.8)
North America	97 (1.9)
Central & South America	67 (1.3)
Australia	63 (1.3)
Eastern Europe	35 (0.7)
Asia	20 (0.4)
Africa	2 (< 0.1)

### Delivery Time Frame

**FIGURE 3:**
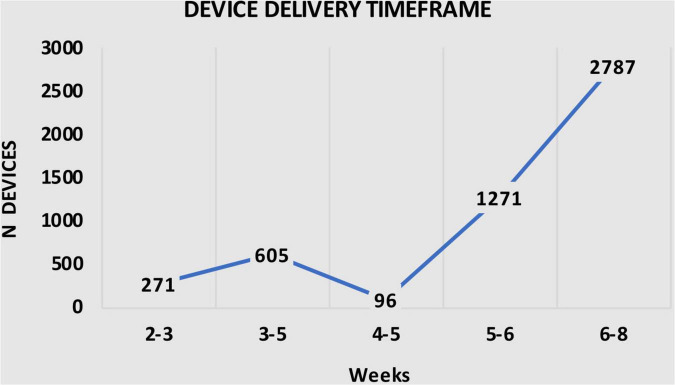
Illustration of device according to delivery time frame.

It is important to note that, as demonstrated in Table 3, all 5030 Anaconda™ devices were delivered within 8 weeks after the diagnosis, with 271 (5.4%) delivered within 2–3 weeks, 605 (12.0%) devices delivered with 3–5 weeks, 96 (1.9%) devices within 4–5 weeks, 1,271 (25.3%) devices within 5–6 weeks, and 2,787 (55.4%) devices within 6–8 weeks after diagnosis (Table 3). Figure 3 shows the number of devices delivered within the time frames.

**TABLE 3:** Summary of device delivery time frames.

Delivery timeframe	n (%)
2–3 weeks	271 (5.4)
3–5 weeks	605 (12.0)
4–5 weeks	96 (1.9)
5–6 weeks	1271 (25.3)
6–8 weeks	2787 (55.4)

### Proximal Ring Stent Configuration

**FIGURE 4:**
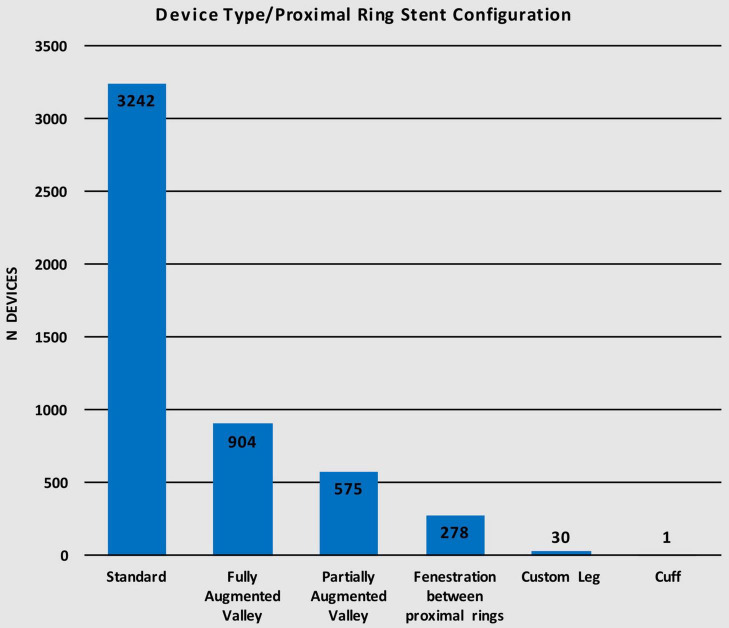
Device type/proximal ring stent configuration.

The Anaconda™ is a highly versatile device offering a wide range of proximal ring stent configurations. Of the 5,030 devices implanted, 3,242 (64.5%) used the standard configuration for the proximal ring while 904 (18.0%) of the devices used the fully augmented valley configuration, 575 (11.4%) used the partially augmented valley, 278 (5.5%) had fenestrations between the proximal rings, 30 (0.6%) utilized a custom leg configuration, and only 1 (< 0.1%) had a cuff configuration (Table 4). Figure 4 illustrates the distribution of devices using each of the six different proximal ring stent configurations.

**TABLE 4:** Device type/proximal ring stent configuration.

Device type/Proximal ring stent configuration	n (%)
Standard	3242 (64.5)
Fully Augmented Valley	904 (18.0)
Partially Augmented Valley	575 (11.4)
Fenestration between proximal rings	278 (5.5)
Custom Leg	30 (0.6)
Cuff	1 (< 0.1)

### Prototype Requirement and Anaconda™ Utilization

**FIGURE 5:**
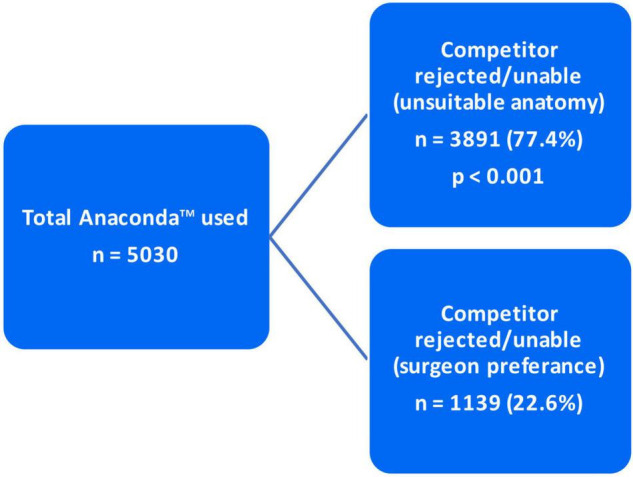
Anaconda™ utilization indication.

**FIGURE 6:**
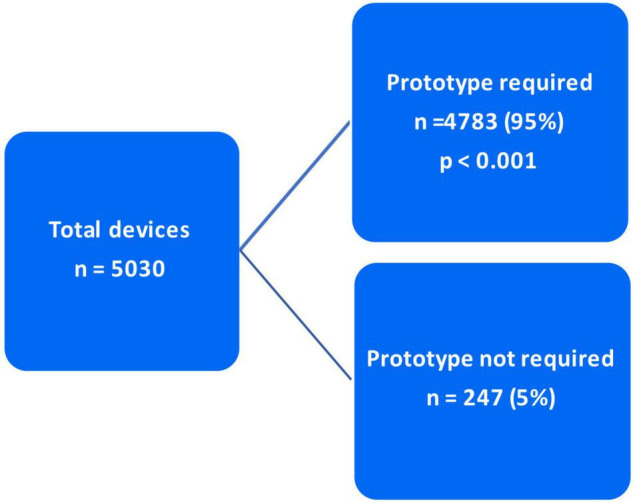
Anaconda™ prototype requirement.

As mentioned in the “Materials and methods” section, the decision to utilize Anaconda™ was made based on two criteria. In 3,891 (77.4%) cases, Anaconda™ was indicated due to unsuitable/complex anatomy for any of the competitor devices. However, in the other 1,139 (22.6%) patients, the Anaconda™ was preferred over the competitor due to surgeon preference (*p* < 0.001) (Figure 5). Of the total 5,030 devices deployed, 4,783 (95%) devices were delivered with a prototype device while 247 (5%) were delivered without a prototype (*p* < 0.001) (Figure 6).

### Number of Fenestrations Used

**FIGURE 7:**
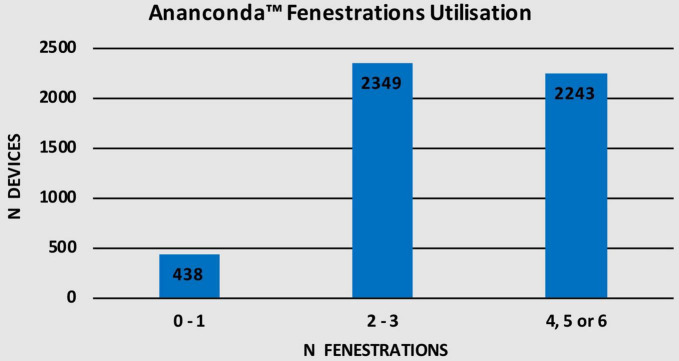
Number of fenestrations used.

Given that the fenestrated Anaconda™ is a CMD, different number of fenestrations can be employed as illustrated in Table 5. Up to 1 fenestration was used in 438 (8.7%) devices while 2,349 (46.7%) devices were used with 2–3 fenestrations. Additionally, 2,243 (44.6%) devices were used with either 4, 5, or 6 fenestrations. Figure 7 shows the number of devices in each fenestration subgroup.

**TABLE 5:** Number of devices used according to number of fenestrations used.

Number of fenestrations	Anaconda™ utilization n (%)
0–1	438 (8.7)
2–3	2349 (46.7)
4, 5 or 6	2243 (44.6)

## Discussion

As indicated in the “Materials and methods” and “Results” section, out of the 5,030 Anaconda™ devices implanted globally, 3,891 (77.4%) were utilized due to the competitors’ inabilities/incompatibilities to treat the unsuitable/complex aortic anatomy presented. The third-generation Anaconda™ innovative design features, along with its unique custom-made approach, allow it to thrive in hostile aortic anatomy. In addition, the Anaconda™ IFU allows a wider range of indications compared to competitors, which enables it to be used in tortuous clinical circumstances where other devices fail. The Anaconda™ is also a highly versatile endograft offering a wide range of device categories and configurations (15–17). The availability of the device prototype has been proven to be a highly valuable tool in the era of custom-made endografts. This is evident in Taher et al. (19), who reported that 21.7% of their 60 Anaconda™ devices were modified after prototype testing. As demonstrated in our results, 95% of the 5,058 Anaconda™ devices implanted were delivered along with a prototype. Similarly, Pini et al. (20) were provided with Anaconda™ prototypes for all their 127 cases.

The Anaconda™’s superiority in rough terrain as mentioned earlier is evident in a multicenter 6-year prospective cohort study by Rodel el al. (21). The study population consisted of 36 patients (83.3% men) with infrarenal AAAs with severely angulated necks (> 60°) treated with the second-generation Anaconda™. The mean neck angulation was 82°, which is outside the IFU of many devices, including Zenith. Successful deployment was achieved in 34/36 (94.4%) patients while the primary technical success rate was 83.3%. After 30 days, all-cause mortality, primary clinical success, primary assisted and secondary clinical success rates were 0, 88.9, and 94.4%, respectively. This demonstrates the excellent performance of the Anaconda™ even in higher-risk clinical circumstances. During the full study period, no aneurysm-related deaths occurred. Freedom from reintervention was 89, 83, and 80% at 1, 2, and 3 years of follow-up. Similarly, freedom from endograft migration was 100 and 97% after 2 and 4 years, respectively (21). These rates are superior to freedom from reintervention and endograft migration achieved with Zenith (22–25). Interestingly, incidence of iliac limb occlusion was 14%, which is still comparable to values reported in studies using other devices in normal circumstances (21). For example, in their Zenith group, Bogdanovic et al. reported a 12.4% rate of limb occlusion (26).

The French multicenter prospective observational study (EPI-ANA-01) by Midy et al. (27) used the Anaconda™ endograft in 176 patients with a suitable infrarenal AAA. However, 33.9% had hostile neck anatomy (with neck angulation >90° in 5.1%) and 10.7% were treated outside the already widespan IFU. Hostile neck anatomy was defined as any or all of length < 10 mm, angle > 60°, diameter > 28 mm, ≥ 50% circumferential thrombus, ≥ 50% calcified neck, and reverse taper. Successful deployment was achieved in 99.4% of patients with 98.3 and 94.9% technical and clinical success rates, respectively. The repositionable deployment system was used in 36.4% of the cases (31.7% in hostile necks vs. 33.3% in favorable necks; *p* = 0.87) and the magnet wire cannulation system in 93.8%. All Anaconda™ grafts were bifurcated, and all three limb configurations were utilized (32.4% straight, 2.8% tapered, 64.8% flared). Importantly, outstanding clinical outcomes were achieved, including 1.7% 30-day mortality, 4.3% limb occlusion, and 19.9% secondary intervention and significant aneurysm sac regression (27). These results, despite the technical and clinical challenges, are superior to those achieved with Zenith. Oderich et al. (23) and Vaaramaki et al. (24) reported a 29.9 and 22% reintervention rate, respectively, at 12 months post-EVAR using Zenith. The latter study also observed a 5% incidence of iliac limb occlusion (24).

Demanget and colleagues (14) modeled eight commercial EVAR devices using finite element analysis. The grafts’ flexibility was assessed, and a numerical benchmark combining bending up to 180° and pressurization at 150 mmHg was performed. Owing to its circular-stented design, the Anaconda™ demonstrated the lowest luminal reduction rate as well as the lowest maximal stress both at 180°, hence greater flexibility and durability compared to the other grafts, particularly the Z-stented grafts, including Zenith (14). Furthermore, using a custom-developed testing apparatus, Crawford investigated the structural impact of misaligned fenestrations in Anaconda™ and Zenith regarding luminal patency and proximal aortic neck apposition (28). The Anaconda™ demonstrated a linear decrease in luminal patency as the angulation degree was increased without any losses in wall apposition, while in contrast Zenith was associated with a decrease in cross-sectional area along with a significant loss in wall apposition (28). Therefore, it can be suggested that the mechanical properties of the Anaconda™ make it a more effective device for extremes of the vascular anatomy.

**FIGURE 8:**
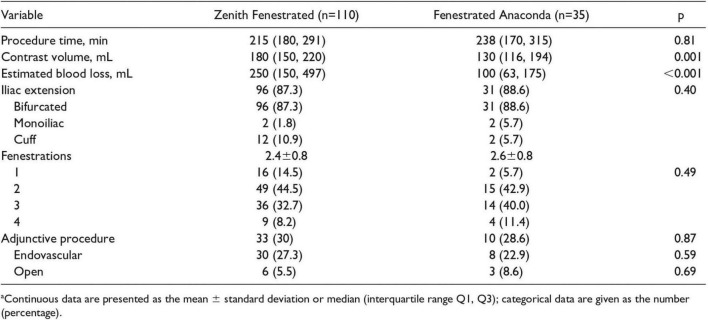
Procedural details of FA and ZP endografts implantations. Reproduced from de Niet et al. (29).

Finally, de Niet et al. (29) also compared Anaconda™ against Zenith. This 14-year study of 145 patients sought to evaluate the conformability of both the Anaconda™ (*n* = 35) and Zenith (*n* = 110) endografts. The influence of different graft designs on aortic anatomy after implantation was assessed. The procedural details of both devices can be seen in Figure 8 [reproduced from de Niet et al. (29)]. The slight difference in procedural time was insignificant, but, on the other hand, both contrast volume and estimated blood loss were significantly less with the Anaconda™ graft. In addition, the majority of devices used were bifurcated and with two fenestrations in both groups. Interestingly, 36 adjunctive procedures were required in 33 Zenith patients compared with 11 in 10 Anaconda™ patients, shedding light on the Anaconda™ more advanced delivery system (29).

## Conclusion

There is enough evidence to suggest that the Anaconda™ is associated with superior performance and optimal delivery thanks to its distinctively novel design. This, in addition to its custom-made approach and high versatility, makes it the prime endograft choice to navigate tortious aortic anatomy where competitors will not dare to or simply cannot.

## Data Availability Statement

The original contributions presented in this study are included in the article/supplementary material, further inquiries can be directed to the corresponding author.

## Ethics Statement

Ethical review and approval was not required for the study on human participants in accordance with the local legislation and institutional requirements. Written informed consent for participation was not required for this study in accordance with the national legislation and the institutional requirements.

## Author Contributions

MJ, AS, and ST were involved in the drafting of the manuscript. DB, IW, and MB were involved in reviewing and providing feedback on the manuscript. All authors contributed to the article and approved the submitted version.

## Conflict of Interest

On behalf of the Southeast Wales Vascular Network (DB and IW) and National Cardiovascular Research Network (DB). The remaining authors declare that the research was conducted in the absence of any commercial or financial relationships that could be construed as a potential conflict of interest.

## Publisher’s Note

All claims expressed in this article are solely those of the authors and do not necessarily represent those of their affiliated organizations, or those of the publisher, the editors and the reviewers. Any product that may be evaluated in this article, or claim that may be made by its manufacturer, is not guaranteed or endorsed by the publisher.
